# Preoperative HbA1c and Blood Glucose Measurements in Diabetes Mellitus before Oral Surgery and Implantology Treatments

**DOI:** 10.3390/ijerph20064745

**Published:** 2023-03-08

**Authors:** Dániel Végh, Bulcsú Bencze, Dorottya Banyai, Adam Vegh, Noémi Rózsa, Csaba Nagy Dobó, Zita Biczo, Gabor Kammerhofer, Marta Ujpal, Leonardo Díaz Agurto, Ignacio Pedrinaci, Juan Francisco Peña Cardelles, Gabriel Leonardo Magrin, Ninad Milind Padhye, Laura Mente, Michael Payer, Peter Hermann

**Affiliations:** 1 Department of Prosthodontics, Semmelweis University, 1088 Budapest, Hungary; 2 Division of Oral Surgery and Orthodontics, Department of Dentistry and Oral Health, Medical University of Graz, Billrothgasse 4, 8010 Graz, Austria; 3 Department of Pedodontics and Orthodontics, Semmelweis University, 1088 Budapest, Hungary; 4 Department of Oral Diagnostics, Semmelweis University, 1088 Budapest, Hungary; 5 Department of Maxillofacial and Oral Surgery, Semmelweis University, 1088 Budapest, Hungary; 6 Faculty of Dentistry, Postgraduate School, Universidad de Chile, Santiago 7520355, Chile; 7 Department of Oral and Maxillofacial Surgery, Plastic Surgery, University Medical Centre, Johannes Gutenberg-University, 55131 Mainz, Germany; 8 Section of Graduate Periodontology, Faculty of Dentistry, Complutense University of Madrid, 28040 Madrid, Spain; 9 Department of Restorative Dentistry and Biomaterials Science, Harvard School of Dental Medicine, Harvard University, Boston, MA 02115, USA; 10 Science Committee for Antibiotic Research of Spanish Society of Implants (SEI—Sociedad Española de Implantes), 28020 Madrid, Spain; 11 Department of Basic Health Sciences, Rey Juan Carlos University, 28922 Madrid, Spain; 12 Oral and Maxillofacial Surgery Department, School of Dental Medicine, University of Connecticut Health, Farmington, CT 06030, USA; 13 Center for Education and Research on Dental Implants (CEPID), Department of Dentistry, Federal University of Santa Catarina (UFSC), 88040-900 Florianopolis, Brazil; 14 Centre for Oral Clinical Research, Queen Mary University and The London School of Medicine and Dentistry, The Royal London Dental Hospital, London E1 1FR, UK

**Keywords:** diabetes, HbA1c, implantology, blood glucose

## Abstract

Diabetes mellitus has become a worldwide epidemic and is frequently accompanied by a number of complications proportional to the duration of hyperglycemia. The aim of this narrative review is to assess the most up-to-date guidelines on DM provided by both diabetes and dental associations. Furthermore, to gather evidence on the uni/bidirectional relationships of elevated HbA1c levels on dental surgery, implantology, bone augmentation, and periodontology and to demonstrate the importance of measuring HbA1c levels before invasive dental treatments. HbA1c and blood glucose measurements are a minimally invasive method for preventing complications in diabetes mellitus. The authors conducted a literature review to determine which oral conditions are affected by diabetes mellitus. MEDLINE served as a source with the use of a specific search key. Regarding oral complications of diabetes, prevention is the most vital factor. With this publication, we hope to assist physicians and dentists to make prompt diagnoses and to help in recognizing various oral manifestations of diabetes and follow the existing guidelines.

## 1. Introduction

Diabetes mellitus (DM) is a global non-communicable disease (NCD). There are more than 500 million people living with DM according to the IDF Atlas statistics [1]. There are various aspects and levels of DM care, which are very important to accomplish, such as therapeutic goals and avoiding long-term complications. Costs of DM-related complications were estimated at $327 billion every year, and values could even be higher, since a large number of people with the disease remain undiagnosed. According to the American Diabetes Association, this expenditure translates to every fourth health care dollar that goes to DM care [2]. Interestingly, more people visited dentists (67.2%) than primary care physicians (52.2%) in the United States (US) according to the National Ambulatory Medical Guidelines in 2014 [3].

Since the dental examination is part of DM care, dental associations provide treatment guidelines for patients with DM. These guidelines focus on annual dental check-ups and a “therapy target” for glycemic levels, categorized by different strategies. If DM is under control, repercussions to the oral cavity have no significant prevalence as compared with a non-DM population [4]. Randomized controlled trials showed that glycemic control can reduce DM complications, which is a key point for DM management [5]. Most authorities have recommended an HbA1c level of <7.0% for DM control, based on the results of the Diabetes Complications and Control Trial (DCCT) [6]. The United Kingdom Prospective Diabetes Study (UKPDS) [7] demonstrated that intensive glucose control substantially reduced onset and delayed progression of microvascular complications. (Australian Diabetes Society Positions Statement) [8].

The scientific literature thoroughly describes and documents the bidirectional relationship between HbA1c and dental complications. Those with better tooth-brushing self-efficacy had lower HbA1c levels and lower plaque scores than those with poor oral hygiene [9,10]. Therefore, methods to encourage preoperative HbA1c testing are essential for patient diagnosis and follow-up. Patients with DM who successfully managed their gingivitis and by undergoing periodontal treatment exhibited better glycemic control and had a lower HBA1c level than those who did not, with a decrease of approximately 0.5% in HbA1c levels [11,12]. On the other hand, there is recent study which contradicts the effect of periodontal therapy on HbA1c levels. The study conducted by Kim et al. found that there was no statistically significant difference between the HbA1c levels of the intervention and control group, although the group receiving the periodontal therapy could maintain their baseline HbA1c levels under the follow-up period, while the control groups’ steadily increased [13]. In light of the most recent diabetology and dental guidelines, the purpose of this review is to present the most up-to-date and effective methods for assessing HbA1c levels before implant treatment and oral surgery, important complications in relation with high blood glucose, and the most important glycemic targets suggested by diabetes and dental associations.

### 1.1. What Is HbA1c

HbA1c is a valuable diagnostic tool for monitoring long-term glycemic control, over approximately 3 previous months. The performance of the test is generally excellent for National Glycohemoglobin Standardization Program (NGSP)-certified assays [14].

### 1.2. HbA1c Methods

#### 1.2.1. Laboratory Analysis

It is possible to determine the serum level of HbA1c by means of a blood test. This process can vary, but usually requires blood collection every 2–3 months, which is the average half-life span of erythrocytes. The majority of technologies used in the laboratory measurement of HbA1c are available for tools used in Point of Care analysis (e.g., boronate affinity chromatography) [15,16].

#### 1.2.2. Point of Care Analysis

POC analysis is a self-testing method for HbA1c level estimation. Most POC devices for HbA1c use a drop of capillary blood collected via the finger-prick procedure. A few minutes after introducing the blood sample to the cartridge, the device analyzes the structure of the hemoglobin. There is a wide variety of technologies which are already available for POC analysis from chromatography to immunoassay techniques (Table 1) [17,18].

### 1.3. Why Is It Important to Monitor HbA1c in the Dental Office?

Intensive glycemic control significantly decreases microvascular complications in patients with short-duration type 2 DM (the Kumamoto Study and the UK Prospective Diabetes Study) [19,20]. Achieving HbA1c targets of <7% (53 mmol/mol) has showed to reduce microvascular complications of type 1 and type 2 DM when instituted early in the course of the disease [21,22].

### 1.4. How Do You Reduce Your HbA1c Level?

The patient and staff of the health care delivery system can control the HbA1c level in numerous ways by utilizing the appropriate type of medication. If necessary, the DM treatment must be modified. Regular exercise can significantly reduce plasma glucose levels. In addition to maintaining a balanced and healthy diet, patients should quit smoking. The most important DM-related information can be found on online platforms. Patients should also have their plasma glucose levels monitored on a regular basis (e.g., Diabetes UK) [23].

### 1.5. Limitations of the Measurement of HbA1c

Exams for HbA1c can produce invalid results due to quantitative and qualitative hemoglobin disorders. Due to their pathologic disintegration, erythrocytes’ lifespan can be shortened, resulting in an HbA1c level that is deceptively lower. Conversely, when the lifespan of erythrocytes is lengthened, a higher HbA1c value can be obtained, which is also present in iron-deficiency anemia. In cases of gestational DM, blood loss and hemodilution can also affect HbA1c values. Hemoglobin variants observed in hemoglobinopathies can result in misleading HbA1c values as well as severe kidney and liver diseases. Since pathological conditions significantly alter the half-life of erythrocytes, conventional methods of monitoring blood glucose levels or fructosamine measurements can be used in place of the HbA1c level [24,25]. In order to detect comorbidities that influence the HbA1c serum level and, consequently, the DM treatment, a thorough medical history is essential.

### 1.6. Prediabetes

Prediabetes (PD) is a condition that exists between the states of health and DM. Patients’ blood glucose levels in PD do not reach the criteria of DM, but they already developed an abnormal carbohydrate metabolism. The HbA1c interval for this condition is considered to be between 5.7 and 6.4%, and it is one of the risk factors for progressing into DM. PD is usually accompanied with obesity, high triglycerides, and hypertension. It is very important to detect patients with undiagnosed PD to interrupt the progression into true DM [26].

An innovative DM screening method that uses gingival crevicular blood (GCB) and could be used to check HbA1c during the periodontal visit is being investigated in a pilot study from New York University. This study’s objective was to look into any undiagnosed DM among periodontal patients. They learned that this is a promising opportunity for public healthcare in detecting patients with undiagnosed DM [27].

## 2. Methods

We concentrated on the guidelines of oral surgery/implantology, periodontology, DM, and their patient associations. An electronic search was conducted to acquire relevant scientific papers to address the research topic. For relevant articles (i.e., human observational studies, randomized/controlled trials, systematic reviews, meta-analyses, and guidelines), the MEDLINE database was screened in January 2023, and no language filters were applied. For the literature search, we used the following search key: (Diabetes Mellitus OR DM OR Type 1 OR Type 2 OR IDDM OR NIDDM OR glycemic target OR glycemic guidelines) AND (HbA1c OR A1c OR Hemoglobin A1c OR glycated hemoglobin) AND (dental implant OR oral surgery OR periodontal therapy OR bone regeneration OR implantology). We were seeking the answer of the effect of DM and different glycemic controls on dental implant surgery, oral surgery, the effect of periodontal treatment on glycemic control, guidelines of DM, and its control.

## 3. HbA1c and Non-Surgical Periodontal Therapy

Non-surgical periodontal treatments, such as professional oral hygiene treatment or root surface scaling, can help reduce the patients’ HbA1c levels [28]. In this topic, a comprehensive review of meta-analyses was conducted, where they sought the answer to the question of whether periodontal treatment improves glycemic control in patients with type 2 DM. A statistically significant decrease in HbA1c levels was found in patients who underwent non-surgical periodontal treatment. Plaque and biofilm removal are routine dental interventions through which the dentist can contribute to the enhancement of the patient’s glycemic control. The review concludes that periodontal treatment should be a routine intervention to improve the glycemic control of patients with DM [29]. However, in the case of patients with DM with advanced chronic periodontitis, the reduction of HbA1c levels was not confirmed. The nonsignificant changes of HbA1c levels between the intervention and control group could be explained with their relatively good glycemic control and on average obese sample population which maintains the higher inflammatory mediators [30]. Another study agrees with this assumption where patients who had high HbA1c levels, therefore poor glycemic control, and had earlier stages of periodontitis benefited more from non-surgical periodontal therapy presenting lower HbA1c levels [31].

In another study, adult patients with type 2 DM were included, who suffered from mild or moderate periodontal disease and were under medical treatment. After root surface scaling and rinsing with mouthwash containing chlorhexidine (0.12%), which was prescribed for the patients to use, a significant decrease in HbA1c level was detected after a 6-month follow-up period [32].

A randomized clinical study conducted by Rapone et al. compared the impact of intensive periodontal treatment (IPT) and supra-gingival mechanical debridement on the endothelial function and lipid-profile of patients with DM. DM can cause insulin resistance through lipotoxicity by reducing the activity of endothelial NO synthase. After the 6 months follow-up, there were no statistically significant difference between the groups in terms of lipid profile and endothelial function, on the other hand patients receiving IPT presented lower HbA1c levels and significantly lower c-reactive protein values. The author suggests that future research with higher sample size is needed in order to clarify the impact of IPT on such outcomes [33].

As for a summary of this section, we can state that medically treated DM with an appropriate intraoral hygiene can contribute towards the improvement of the patient’s glycemic control.

## 4. Tooth Extraction in Patients with DM

In view of the detailed and general dental anamnesis, it is advisable for the dentist, particularly the oral surgeon, to take some important steps before invasive procedures. The possibility of complications, which concern the process of wound healing, is greater in this group of patients. A chronic hyperglycemic state induces the formation of advanced glycation end products in the tissues that impair the chemotactic and phagocytic function of polymorphonuclear leukocytes. This results in the formation of destructive inflammatory cytokines causing delay in the healing process [34]. The majority of oral surgeons are uncertain of the threshold limit of HbA1c or blood glucose levels from where it poses an elevated risk in developing harmful complications [35].

A systematic review was conducted on the topic to find the limit of glycemic control where the risk of developing certain complications for tooth extraction becomes significant. The safe limit of blood glucose levels is 180 mg/dL (during fasting), and the critical limit of blood glucose is the value of 240 mg/dL (during fasting), above which value postoperative complications, for instance, super-infection or delayed socket healing, should be considered. It is advised to utilize antibiotic prophylaxis for patients with poorly controlled DM [36].

## 5. DM-Related Complications—Implant Therapy and Sinus Lift

There is a close relationship between DM and peri-implant inflammation. A meta-analysis, based on the results of several types of studies, has shown that the risk of peri-implantitis in people with DM is 50% higher compared to the systematically healthy group. A statistically significant difference was found since peri-implantitis is 3.39× more frequent in non-smokers with high glycemic levels compared to patients with a standard glycemic value. On the other hand, the connection between peri-implant mucositis and DM is not confirmed. Extra care should be taken while providing dental treatment for patients with DM, such as limiting the presence of periodontal and periapical infections and other systemic conditions which may affect the success of the dental treatment. Currently, antibiotic treatment and anti-infective measurements are routine procedures after the surgical placement of the dental implant, which can help avoiding any possible infectious complications in the critical healing period [37,38].

Individual consideration is required for each patient. In the case of controlled DM, comprehensive research has shown that dental implants can be used safely, which means that osseointegration comes into existence. The usage of antiseptic oral rinses further improves the chances. The HbA1c level, as mentioned above, which should be in an optimal range, and glucose level are similarly authoritative because of the relationship between oral implants and DM [39].

The survival rate of dental implants in people who suffer from controlled type 2 DM is between 92.3–92.4% [40]. Other research has shown 92.6% and 95% survival rates in well-controlled and poorly controlled cases, respectively [41]. The glycated hemoglobin value is excellent for monitoring glucose levels; currently, this value is also considered a diagnostic marker. Dental implantation for patients with high glycemic levels can be performed after individual consideration and by paying increased attention to the individual’s motivation. In that case, the most minimally invasive and tissue-protective surgery is required.

According to the report of the American Diabetes Association (ADA), it is advisable to keep the HbA1c level of patients who suffer from DM under the value of 7% [14]. According to this analysis, the only disadvantage of the HbA1c level is that it is not a cost-effective method, and it requires some technical knowledge. In the case of type 2 DM, Oates et al. consider it a well-controlled case if the value is between 6–8%, a medium well-controlled case if the value is between 8.1–10%, and a poorly controlled case if the value is above 10%. In terms of implant stability, it was significantly affected by the HbA1c value of the patient at the time of the surgical placement. The decrease in implant stability from the time of the placement was significantly greater in medium well- and poorly controlled cases. The time requirement for the implant stability to return to baseline was close to double in these cases, compared to healthy and well-controlled cases [42].

Confirming the results of the previously mentioned studies, a systematic review conducted by Wagner et al. found that patients with poorly controlled DM suffer more from peri-implantitis and implant loss, while the results of patients with good glycemic control are comparable to systematically healthy individuals. The author also highlights the importance of perioperative anti-infective therapy, with the likes of chlorhexidine and antibiotics [38].

There are several types of diseases where using the sinus lift technique means a higher risk [43]. For example, such a systematic disease is DM, which can lead to unfavorable wound healing. The implantation, which happens by using the sinus lift technique, is a more invasive intervention, which means that the likelihood of postoperative complications also increases. Using shorter implants as a compromised treatment plan is often advisable for these groups of patients to avoid sinus lift surgery [44].

HbA1c levels are important not only for implant surgery, but also for long-term implant maintenance and success. A study by Moreno et al. in 2015 showed that there was higher peri-implant mucositis and marginal bone loss over a 2–5-year period for implants placed in subjects with high HbA1c levels. Thus, dental implants must be considered in subjects consistently showing low to moderate HbA1c levels [45].

As for a summary, poor glycemic control poses as a threat for many invasive dental treatments and it elevates the risk of developing adverse complications, therefore extra caution must be taken in such cases (Table 2).

## 6. Current Guidelines of Diabetes Associations—HbA1c

### 6.1. American Diabetes Association—Glycemic Targets—2021

The ADA guidelines first assess the importance of yearly glycemic or HbA1c measurements, and they suggest it two times a year for patients who meet their glycemic treatment goals and possess stable glycemic control. For patients who do not meet their treatment goal or whose therapy has recently changed, quarterly measurements are needed according to the Canadian Diabetes Association [46]. Well-controlled glycemic status is considered to be under 7% HbA1c (53 mmol/mol) in non-pregnant adults without any significant hypoglycemia. A parallel goal can be >7% of time in range and <4% of time below range in case of using ambulatory glucose profile/glucose management indicator. Aiming for a lower HbA1c level than 7% may be beneficial (e.g., <6.5% (64 mmol/mol)) if it can be achieved without any significant hypoglycemia or other adverse side effects on the basis of provider judgment and the patient’s preference. Usually, this is achievable in patients with recent diagnosis of type 2 DM. On the other hand, less strict HbA1c goals, such as below 8% (64 mmol/mol) may be more appropriate for patients with limited life expectancy, or where the harms of the treatment are greater than its benefits [14].

The aforementioned glycemic targets are susceptible to many personal attributes, therefore there are different targets for different age groups and health statuses. For healthy older adults, the target HbA1c level changes to 7.5%, in the case of intermediate health to 8.0%, and in case of poor health to 8.5% [47]. An interesting fact is that pregnant women experience increased red blood cell turnover, which causes a lower average HbA1c level compared to non-pregnant women. In pregnancy, a safe glycemic target is considered to be 6% HbA1c but can be relaxed to 7% to prevent hypoglycemia. Under 6.5% HbA1c, the probability of developing congenital anomalies is identical to that of pregnancies without DM [48]. In case of children, an HbA1c goal of <7% is appropriate for many of them, while in adolescence and young adulthood even lower levels are associated with lower risk of micro- and macrovascular complications [49].

### 6.2. A Consensus Report by the American Diabetes Association (ADA) and the European Association for the Study of Diabetes (EASD) 2018

In the report, suggestions for glycemic treatment targets for nonpregnant adults with sufficient life expectancy (~10 years) were defined to achieve microvascular benefits, which is set to around 7% (53 mmol/mL) or less. It is important to emphasize that this target should be individualized to the patients’ goals, risk of adverse effects (e.g., hypoglycemia, weight gain), patient characteristics, and comorbid conditions [50].

### 6.3. Position Statement by the Australian Diabetes Society (ADS) on the Individualization of HbA1c Targets for Adults with Diabetes Mellitus

In the ADS recommendations, suggestions are made for the glycemic treatment targets of <7.0% HbA1c for most adult patients. This should be individualized to either a tighter or a looser degree, with a recommended target of HbA1c level of <6.0%, or up to <8.0% in other patients [8].

## 7. Current Guidelines of Dental Associations—HbA1c

Dental associations barely take a stand on the expected HbA1c levels of patients undergoing implantology treatment. We found articles on this topic in the literature, but most of them report on a short-term follow-up, and their results are controversial. Some studies suggest no significant difference in implant success among people living with or without DM, not even if their blood glucose level is poorly controlled [41,51]. A recent meta-analysis proved increased probing pocket depth, bleeding on probing, and mobility in implant patients living with type 2 DM after a 12-month follow-up despite having good glycemic control [52].

There was a study with a two-year follow-up period. In this topic, the participants were divided into three groups based on their HbA1c levels. The HbA1c level in the first group was below 6%; in the second group, it was between 6.1–8%; and in the third group, it was between 8.1–10%. One year after the surgery, all three groups had high implant survival rates: in the first two groups, it was 100%, and in the third, it was 95.4%. After two years of follow-up, the implant survival rates among people with the worst controlled glycemia sank to 86.3% due to peri-implant complications [53].

We found one article with a long-term, 8-year follow-up. In this paper, the authors stated that the implant survival rate among people with DM was 95.1% and did not significantly differ from the healthy population. As a limitation of this article, we must mention that HbA1c levels were not noted [54].

It is very important to emphasize the vitality of regular dental recalls in order to avoid any peri-implant inflammation. It is suggested to provide professional dental prophylaxis for patients with DM biannually to significantly reduce the incidence of peri-implantitis and peri-implant mucositis [55].

We believe further investigation, including long-term clinical trials, is still needed. Furthermore, we would like to draw attention to the fact that we must make a difference between implant survival and success. Under implant survival, we understand that the patient did not lose the implant. However, the implant case is considered successful if the implant is functioning properly without inflammatory peri-implant complications [56].

In the periodontology field, it is much more revealed what the expected HbA1c levels are. The new classification of periodontal diseases grades periodontitis in terms of HbA1c levels. If the patients suffer from DM but their HbA1c is under 7%, the grade will be upgraded to “B”; over 7% it will be upgraded to “C” [57].

### DGI, DGZM Guidelines

The German Society of Dentistry and Oral Medicine (DGZM) and the German Association of Implantology (DGI) stipulated in their guidelines that the therapeutic goal for hemoglobin HbA1c should be between 6.5% and 7.5%. They have distinguished three groups based on glycemic control:

- Good glycemic control is associated with an HbA1c level between 6–8%
- Moderate glycemic control is associated with an HbA1c level between 8–10%
- Poor glycemic control is associated with an HbA1c level above 10% [58].

Overall, the studies above emphasize the prompt resolution of comorbid conditions, such as obesity, hyperlipidemia, hypertonia, and a lifestyle burdened by smoking. In the case of initial DM, patients should undergo more intensive and stringent glycemic control. The target value should be determined individually and based on the conditions of the various patients. Several randomized and controlled studies have demonstrated that this disease’s complications can be mitigated through the application of appropriate glycemic control. This narrative review aimed to provide an overview of the significance of HbA1c during dental treatments for DM patients.

According to substantial evidence, lower HbA1c levels were associated with reduced onset or progression of certain microvascular complications [5].

As the guidelines indicate, oral health examination should be an integral part of DM care. There is support for this recommendation. With this review, we aimed to provide evidence supporting preoperative HbA1c measurement for oral health examinations.

It is possible to look at this level based on labor results, if necessary, but with the development of diagnostic tools, it is also possible to use point-of-care machines. It is important to take into account the glycemic control recommendations of major associations, in order to avoid adverse complications (Table 3).

## 8. The Future of the Disease—WHO Global Targets for DM

In 2022, the WHO began an ambitious project by creating the first global targets for DM which is aimed to be achieved by 2030. The main principles of these targets are the following:

- 80% of the people with DM are diagnosed
- 80% of the diagnosed patients have good glycemic control and blood pressure
- 60% of these patients above the age of 40 will receive statins
- 100% of the patients with type 1 DM will have access to insulin and self-monitoring of blood glucose levels

The impact of these targets could be numerous. First and foremost, it would decrease the prevalence of acute complications and mortality. It would increase the overall population health outcomes and could lead to lower health-care costs. Moreover, a healthy population can contribute to the economy in a higher quality for a longer period of time [59].

Dental practitioners can contribute in a meaningful way towards the achievement of these global targets by detecting patients with undiagnosed DM with the help of routine chair-side HbA1c measurements. Since poorly controlled DM is a significant risk factor for many dental procedures, encouraging these patients to manage their glycemic control with the help of their diabetologist is a must to avoid unnecessary dental complications.

## 9. Limitations of the Study

This review had several limitations, but the authors wanted to highlight the importance of this critical selection of patients and also to measure the possible presurgical complication factors to avoid the long-term side effects of poorly controlled DM. The definition of poorly controlled DM is not an exact number or marker, as different organizations measure different values. We will need a possible consensus between diabetologists and dentists, such as the Madrid consensus, to provide a clear guideline for the general dentist to follow in case of surgical treatments in the oral cavity or placing implants with or without sinus elevations [60].

## 10. Conclusions

Diabetes associations advise certain glycemic targets for people with DM; these target values (most commonly <8% HbA1c) should be a core message to oral health providers. As dental associations suggest, annual or more regular oral examinations and the registration or measurement of current glycemic status are recommended for patients with DM to avoid the disease’s long-term complications, such as implant loss, impaired bone and wound healing, and increased susceptibility to infections.

## Figures and Tables

**Table 1:** List of the most common scientifically supported methods for HbA1c measurements.

Methods for HbA1c Measurements
Cation-exchange chromatography
Immunoassay
Affinity chromatography
Enzymatic assay

**Table 2:** Possible complications of high glycemic levels.

Dental Treatment	Most Important Complications
Periodontal treatment	Bidirectional relationship: higher HbA1c levels manifests in a higher grade of periodontitis, while periodontal treatment could improve the patients HbA1c levels
Oral surgery	Above the critical blood glucose value, the risk of developing surgical complications increases, e.g., alveolitis, delayed healing
Implantology	Poor glycemic control elevates the risk of peri-implantitis, increases crestal bone loss, and peri-implant soft tissue inflammatory parameters

**Table 3:** Important glycemic targets according to the major associations.

Associations	Suggested HbA1c	Good Control	Poor Control
ADA 2021	<7%	<8%	>8%
ADA, EASD 2018	<7%	NA	NA
ADS 2009	<7%	<8%	>8%
AAP, EFP 2017	NA	<7%	>7%
DGI, DGZM 2016	6.5–7.5%	<8%	Moderate: 8–10% Poor: >10%

ADA: American Diabetes Association; EASD European Association for the Study of Diabetes; ADS: Australian Diabetes Society; AAP: American Academy of Periodontology; EFP: European Federation of Periodontology; DGZM: German Society of Dentistry and Oral Medicine; DGI: German Association of Implantology.

## Data Availability

The datasets used and/or analyzed during the current study are available from the corresponding author on reasonable request.

## Abbreviations

DM	diabetes mellitus
SMBG	self-monitoring of blood glucose
CGM	continuous glucose monitoring
TIR	time in range
FSB	Finger Stick Blood
GCB	Gingival Crevicular Blood
OGTT	Oral Glucose Tolerance Test
WHO	World Health Organization
